# Identification of germplasm and genomic markers associated with reduced T‐2 and HT‐2 mycotoxin accumulation in oat

**DOI:** 10.1002/tpg2.70321

**Published:** 2026-10-09

**Authors:** Farhana Afroze, Natarajan Subramani, Amal Kahla, Cathal McCabe, Angela Feechan, Atikur Rahman, Humberto Fanelli Carvalho, Julio Isidro‐Sánchez, Fiona Doohan

**Affiliations:** ^1^ School of Biology and Environmental Science University College Dublin Dublin Ireland; ^2^ School of Agriculture and Food Science University College Dublin Dublin Ireland; ^3^ Institute of Life and Earth Sciences Heriot‐Watt University Edinburgh UK; ^4^ Crop Science Department Teagasc Oak Park Carlow Ireland; ^5^ Centro de Biotecnología y Genómica de Plantas (CBGP, UPM‐INIA) Universidad Politécnica de Madrid (UPM) ‐ Instituto Nacional de Investigación y Tecnologia Agraria y Alimentaria (INIA) Campus de Montegancedo‐UPM Pozuelo de Alarcón Spain

## Abstract

Harmful T‐2 and HT‐2 mycotoxins are produced by *Fusarium langsethiae* and *Fusarium sporotrichioides* in developing oat (*Avena sativa* L.) grain. This study identified oat genotypes with consistently low toxin accumulation across environments. Toxin enzyme‐linked immunosorbent assay and fungal DNA (quantitative polymerase chain reaction) levels were quantified in 19 oat genotypes grown at two Irish sites under natural infection. *F. langsethiae* and T‐2 + HT‐2 were detected at both sites, while *F. sporotrichioides* was absent. Genotype effects were significant for both *F. langsethiae* DNA and toxin levels, and the traits were positively correlated (Spearman's *ρ* = 0.62, *p* < 0.001). Eleven genotypes were also evaluated in an *F. langsethiae*‐inoculated glasshouse experiment within a larger panel of 190 genotypes. Across field and glasshouse environments, toxin levels were consistently low in Tempo, Sandy, and Jokikyla ME0501 and high in Stormont Arrow. Glasshouse T‐2 + HT‐2 levels ranged from below detection to 1000 µg kg^−^^1^. genome‐wide association study using 4881 single‐nucleotide polymorphisms identified five significant markers on Mrg11, mapping to two loci on chromosomes 6A and 4C associated with reduced toxin accumulation. The 6A locus was near HIPP39 (where HIPP is heavy metal‐associated isoprenylated plant protein), whereas no coding genes occurred within ± 30 kb of the 4C locus. These associations were detected in only one of the two glasshouse runs and differed from loci previously identified under field conditions, highlighting the need for a better understanding of the elusive and variable nature of *Fusarium*‐oat interactions. These results highlight genotypes and candidate loci to support breeding for reduced T‐2 and HT‐2 risk in oats.

## Core Ideas

- Field trials (19 oat genotypes) and controlled glasshouse trials (190 oat genotypes) revealed substantial genetic variation in susceptibility to *Fusarium langsethiae* and in T‐2 and HT‐2 toxin accumulation on oat grain.
- Quantitative polymerase chain reaction confirmed the presence of *F. langsethiae* across both Irish field sites, whereas *Fusarium sporotrichioides* was not detected, identifying *F. langsethiae* as the principal contributor to toxin contamination.
- Several oat genotypes, including Sandy, Tempo, Glasnevin Major, Conway, and Jokikyla ME0501, showed consistently low toxin accumulation.
- Genome‐wide association analysis identified two loci represented by five single‐nucleotide polymorphism markers on linkage group Mrg11, mapping to oat chromosomes 6A and 4C in the OT3098 v2 assembly (both markers fall within a single genotyping‐by‐sequencing consensus linkage group, Mrg11), which were associated with variation in toxin accumulation under controlled inoculation.
- Candidate gene analysis identified HIPP39 (where HIPP is heavy metal‐associated isoprenylated plant protein) as a strong positional candidate on chromosome 6A, whereas the associated locus on chromosome 4C did not co‐localize with annotated genes within the examined genomic window.

## Plain Language Summary

Mycotoxins produced by certain fungi can contaminate oat grain and pose risks to food and feed safety. The fungus *Fusarium langsethiae* commonly infects oats without causing visible disease symptoms, yet it can lead to the accumulation of the harmful mycotoxins T‐2 and HT‐2. In this study, we examined how different oat varieties respond to this fungus under natural field conditions and under controlled glasshouse inoculation. We identified several oat varieties that consistently accumulated very low toxin levels across both environments. Using toxin data from a larger panel of oats grown under controlled conditions, we linked reduction in toxin accumulation to specific regions of the oat genome. These findings show that genetic differences among oat varieties play a key role in determining toxin risk and identify genomic regions that can be used by plant breeders to develop oat varieties with reduced mycotoxin contamination.

**Abbreviations**
ELISA: enzyme‐linked immunosorbent assay
FDR: false discovery rate
FHB: Fusarium head blight
GWAS: genome‐wide association study
HIPP: heavy metal‐associated isoprenylated plant protein
PCR: polymerase chain reaction
qPCR: quantitative polymerase chain reaction
QTL: quantitative trait locus
SNP: single‐nucleotide polymorphism

## 1 INTRODUCTION

In recent years, demand for oat grain has risen sharply, driven by public awareness of its nutritional and health benefits. Oats are a rich source of soluble fiber, protein, antioxidants, vitamins, and minerals (Alemayehu et al., 2023; Rasane et al., 2013). Their cholesterol‐lowering effects, largely attributed to β‐glucan, are well established (Holopainen‐Mantila et al., 2024; Joyce et al., 2019). Oats also provide environmental advantages due to their relatively low input requirements compared with other cereals (Menon et al., 2016).

Fusarium head blight (FHB) is an economically important disease of oats and other small grains, reducing grain quality and yield and contaminating grain with mycotoxins, including deoxynivalenol (DON), nivalenol (NIV), zearalenone, and the type A trichothecenes T‐2 and HT‐2 toxins. Several *Fusarium* species contribute to this disease complex. *Fusarium graminearum* and *Fusarium culmorum* are associated with DON accumulation, *Fusarium poae* with NIV, and *Fusarium langsethiae* and *Fusarium sporotrichioides* with T‐2 and HT‐2 toxins (Dinolfo et al., 2012; Nazari et al., 2016). While FHB symptoms such as premature bleaching and chlorophyll loss are primarily associated with the highly pathogenic *F. graminearum* and *F. culmorum* (Hautsalo et al., 2020; Xue et al., 2015), infections by *F. langsethiae* are typically symptomless (Aamot et al., 2022; Imathiu et al., 2013; Martin et al., 2018). Furthermore, both *F. poae* and *F. sporotrichioides* often produce only mild or inconsistent visible symptoms depending on the genotype and environment (Xue et al., 2015; Schurack et al., 2025).

This hidden contamination poses a direct challenge to food safety. In Ireland, T‐2 and HT‐2 toxins are recurrent contaminants, detected in 41%–61% of oat samples in recent surveys (De Colli et al., 2021; Kolawole et al., 2021). Similar patterns have been reported elsewhere in northern Europe, including Sweden, Finland, and the United Kingdom, where frequent T‐2 and HT‐2 contamination of oat has been documented under natural field conditions (Edwards & Stancic, 2024; Edwards et al., 2012; Fredlund et al., 2010). In response to food safety concerns, the European Union established updated limits in 2024, including a maximum of 1250 µg kg^−^^1^ for combined T‐2 and HT‐2 toxins and 1750 µg kg^−^^1^ for DON in unprocessed oat. While processing (mainly dehulling) can reduce toxin loads, it risks concentrating them in by‐products. Stricter limits apply to consumer‐ready products, being 100 µg kg^−^^1^ for the combined T‐2 and HT‐2 in oats for direct consumption and 600 µg kg^−^^1^ for DON in milled products (European Commission, 2024a, 2024b; Pettersson et al., 2011). Consequently, minimizing toxin accumulation in the field remains the most effective control strategy.

Breeding offers a promising route to minimize toxin accumulation in oats, as genotypes exhibit substantial natural variation in T‐2 and HT‐2 accumulation. Spring‐type oat varieties generally accumulate lower T‐2 and HT‐2 levels than winter‐type varieties (Edwards & Stancic, 2024), while some genotypes show much lower contamination than others (Hofgaard et al., 2022; Martin et al., 2018). For example, in grain analyzed from multilocation field trials across 3 years in Norway, var. Vinger accumulated less than a third of the toxin levels of var. Odal (Hofgaard et al., 2022). Resistance to the accumulation of other *Fusarium* mycotoxins has also been documented in oats; for example, genotypes such as Leggett and Horizon270 carry alleles conferring resistance to FHB and reduced accumulation of DON (Bjørnstad et al., 2017).

To harness this variation for breeding, it is essential to identify the underlying genetic basis of the resistance mechanisms. In wheat, reduced mycotoxin phenotypes are recognized to arise through multiple resistance mechanisms, including reduced infection or pathogen spread (Type I/II resistance), as well as toxin‐focused mechanisms that limit mycotoxin accumulation via toxin modification or detoxification, sequestration, or altered transport within plant tissues, often conceptualized as Type III/IV/V resistance in the FHB literature (Ma et al., 2022; Mesterhazy, 2020). These toxin‐related mechanisms have been a major focus of wheat breeding because they contribute directly to lower mycotoxin levels (including DON) in harvested grain, and genetic loci influencing both disease resistance and toxin control have been identified (Figlan & Mwadzingeni, 2022). However, toxin‐targeted resistance mechanisms remain comparatively underexplored in oat, highlighting a key knowledge gap that genetic mapping approaches such as genome‐wide association studies (GWASs) can help to address.

The identification of genomic loci associated with reduced mycotoxin accumulation can accelerate the development of low‐toxin, FHB‐resistant oat varieties. A GWAS that associated the Irish field response of 190 spring oat varieties to *F. langsethiae* linked five single‐nucleotide polymorphisms (SNPs) and a single quantitative trait locus (QTL) on the merged linkage group Mrg06 with T‐2 and HT‐2 accumulation (Isidro‐Sánchez et al., 2020). The associated marker *avgbs_6K_95238.1* mapped within genes showing similarity to lipases and zinc finger proteins gene families with established roles in plant defense signaling and the pathogen response (Isidro‐Sánchez et al., 2020). Another oat GWAS study identified genetic loci associated with resistance to *F. graminearum* and DON content, delineating six QTLs associated with low DON (Bjørnstad et al., 2017), including three previously reported for low FHB disease or low DON (He et al., 2013).

**Core Ideas**

- Field trials (19 oat genotypes) and controlled glasshouse trials (190 oat genotypes) revealed substantial genetic variation in susceptibility to *Fusarium langsethiae* and in T‐2 and HT‐2 toxin accumulation on oat grain.
- Quantitative polymerase chain reaction confirmed the presence of *F. langsethiae* across both Irish field sites, whereas *Fusarium sporotrichioides* was not detected, identifying *F. langsethiae* as the principal contributor to toxin contamination.
- Several oat genotypes, including Sandy, Tempo, Glasnevin Major, Conway, and Jokikyla ME0501, showed consistently low toxin accumulation.
- Genome‐wide association analysis identified two loci represented by five single‐nucleotide polymorphism markers on linkage group Mrg11, mapping to oat chromosomes 6A and 4C in the OT3098 v2 assembly (both markers fall within a single genotyping‐by‐sequencing consensus linkage group, Mrg11), which were associated with variation in toxin accumulation under controlled inoculation.
- Candidate gene analysis identified HIPP39 (where HIPP is heavy metal‐associated isoprenylated plant protein) as a strong positional candidate on chromosome 6A, whereas the associated locus on chromosome 4C did not co‐localize with annotated genes within the examined genomic window.

The aim of this study was to enhance our understanding of the germplasm, varieties, and genetic basis of low T‐2 and HT‐2 toxin accumulation in oats. The first objective of this study was to build on previous Irish surveys by identifying oat genotypes with consistently low T‐2 and HT‐2 accumulation and *Fusarium* colonization under natural field conditions across two locations in Ireland. We evaluated 19 genotypes at two field sites, and a subset of 11 of these was also assessed under controlled glasshouse conditions with artificial inoculation with *F. langsethiae*. As stated above, a previous field‐based GWAS conducted in Ireland identified QTL associated with T‐2 and HT‐2 accumulation in response to *F. langsethiae* inoculation (Isidro‐Sánchez et al., 2020). The second objective of this study was to repeat this GWAS under glasshouse conditions to determine whether the same or different QTLs are associated with low toxin levels when we remove the influence of fluctuating environmental conditions and natural field inoculum. By inoculating the same panel of 190 oat varieties with *F. langsethiae* under controlled conditions, we isolated the host–pathogen interaction and minimized environmental variation. This approach allowed us to expand the repertoire of markers associated with low toxin accumulation in oats and to assess whether the genomic regions associated with toxin accumulation under field conditions could also be detected when host–pathogen interactions were examined under controlled, standardized conditions, thereby enabling comparison of resistance loci identified across contrasting environments.

## 2 MATERIALS AND METHODS

### 2.1 Plant and fungal materials

A total of 19 oat (*Avena sativa* L.) genotypes, including modern genotypes and heritage varieties, were screened under field conditions for natural infection by *F. langsethiae* and *F. sporotrichioides* and for associated T‐2 and HT‐2 mycotoxin contamination (Table S1). Of these, 11 genotypes were also evaluated under glasshouse conditions for toxin accumulation following *F. langsethiae* inoculation (Table S1). The 190 spring oat genotypes used for the glasshouse GWAS experiment were previously described by Isidro‐Sánchez et al. (2020) and are listed in Table S2. These include breeding lines and varieties that were assessed in earlier field studies for mycotoxin accumulation.

Fungal strains used in this study are listed in Table S3. *F. langsethiae* strain F1201059 (Lysøe et al., 2016) and *F. sporotrichioides* strain 89/N (SBES culture collection, University College Dublin) served as positive controls for species‐specific quantitative polymerase chain reaction (qPCR) assays. The strain used for glasshouse inoculation was *F. langsethiae* Fl201059. Mycelial cultures were revived from −80°C glycerol stocks onto potato dextrose agar (PDA; DIFCO Laboratories) and incubated at 20°C for 7 days in the dark. Conidia were produced in oat broth and harvested following the protocol of Isidro‐Sánchez et al. (2020). Spore suspensions were adjusted to 1 × 10^5^ microconidia mL^−^^1^ in 0.02% Tween‐20, and inoculum was freshly prepared each week during flowering.

### 2.2 Field trials

Field trials of 19 genotypes were conducted in 2022 at two locations in Ireland: UCD Lyons Farm, Co. Kildare (53.489603°N, −6.668142°W), and Teagasc Oak Park, Co. Carlow (52.867154°N, −6.922498°W). Trials were sown in late March and harvested in mid‐August. Each genotype was grown in three replicate plots (10–12 m^2^) in a randomized layout, with sowing density of 300–350 seeds m^−^^2^. Standard agronomic practices were followed, but no fungicides were applied for FHB control. At GS95, panicles were harvested, threshed, and hulled, freeze‐dried, milled, and stored at −20°C for analysis. For mycotoxin quantification, each biological replicate (plot) was analyzed with two technical replicates. Similarly, for qPCR, each biological replicate was analyzed using three technical replicates, and the mean of the three technical replicates was used for statistical analysis.

### 2.3 Glasshouse experiment

A glasshouse experiment was conducted at Rosemount Environmental Research Station, University College Dublin, to assess the response of 190 oat genotypes to *F. langsethiae* inoculation, of which 11 were also evaluated in the field trials. Seeds were germinated in Petri dishes lined with moist Whatman No. 1 filter paper, stratified at 4°C for 48 h, and then incubated at 21°C for 72 h. Seedlings were transplanted into 3 L pots (two plants per pot) filled with John Innes No. 2 compost (Westland Horticulture). Plants received a top‐dress application of slow‐release fertilizer (Osmocote Topdress FT 4–5 M, ICL) at 6 weeks after planting (2 g L^−^^1^) and were watered regularly to maintain consistent soil moisture. Plants were maintained under a 16/8 h light‐dark cycle at minimum temperatures of 18/25°C (night/day), 70% relative humidity, and 300 µmol m^−^^2^ s^−^^1^ light intensity. The experiment comprised two replicated trials conducted from September 2021 to January 2022 and from January 2022 to June 2022. At mid‐anthesis (GS65), two panicles per plant were sprayed to run‐off with either 4 mL of 0.02% Tween 20 (mock) or *F. langsethiae* conidial suspension (1 × 10^5^ microconidia mL^−^^1^) using a hand‐held nozzle sprayer (Hozelock Ltd.). Treated panicles were enclosed in wet polythene bags for 48 h, and the glasshouse floor was irrigated regularly to maintain elevated humidity. At GS91, panicles were harvested and threshed (Almaco Wheat Thresher SVPT). For each genotype in each trial, two composite samples were prepared by pooling seeds from two panicles per treatment. Grain samples were freeze‐dried, milled to a fine powder using a coffee grinder (Bodum), and stored at −20°C until analysis.

### 2.4 Mycotoxin quantification

T‐2 and HT‐2 toxins were quantified in both field and glasshouse grain samples using the Bio‐Shield T‐2 + HT‐2 enzyme‐linked immunosorbent assay (ELISA) kit (ProGnosis Biotech) following the manufacturer's protocol. Approximately 5 g of finely milled oat grain was extracted in 25 mL of 70% methanol (v/v), and the supernatant was collected for analysis. Each sample (biological replicate) was analyzed in two technical replicates, and the mean of the two technical replicates was used for subsequent statistical analyses. Absorbance was measured at 450 nm (Tecan Infinite 200 PRO), and concentrations were calculated from standard curves using ProGnosis Data Reader Software v7.4.1.31. The assay detection limit was ≤20 µg kg^−^^1^; values below this threshold were substituted with 20 µg kg^−^^1^. ELISA results were validated by liquid chromatography‐tandem mass spectrometry at Queen's University Belfast for 11 random glasshouse samples (data not shown), following established procedures (Meneely et al., 2023).

### 2.5 DNA extraction and quantification of *Fusarium* biomass

Genomic DNA was extracted from milled grain (200 mg) and from fungal mycelia (scraped from PDA plates, flash‐frozen in liquid nitrogen, and ground using a mortar and pestle) using the Diatheva Extraction Kit (Diatheva), following the manufacturer's protocol. DNA quality and quantity were determined using a Biodrop µLITE spectrophotometer (Labplan), and samples were stored at −20°C.

Quantification of *Fusarium* biomass in field samples was performed by qPCR using species‐specific assays for *F. langsethiae* and *F. sporotrichioides* on a QuantStudio 7 Flex Real‐Time PCR System (Applied Biosystems). Plant DNA was quantified in parallel as an endogenous control using primers COX554‐f and COX554‐r targeting the mitochondrial cytochrome oxidase (COX) gene, adapted from the published TaqMan assay (Divon et al., 2011) for use with SYBR Green chemistry (Table S4). Assays and primers used for fungi were as previously described (Divon et al., 2011; Nicolaisen et al., 2009). Each 10‐µL reaction contained Fast SYBR Green Master Mix (Applied Biosystems), 300 nM of each primer, and 10 ng template DNA. Reactions were run in triplicate, and the mean of the three technical replicates was used for subsequent statistical analyses. Absolute DNA quantities were calculated from standard curves generated from fivefold serial dilutions (fungal DNA: 1000–16 pg per reaction; plant DNA: 40,000–64 pg per reaction). All assays showed amplification efficiencies between 90% and 110% and coefficients of determination (*R* ^2^) for standard‐curve linear regressions >0.99. Fungal biomass was expressed as picograms of *Fusarium* DNA per nanogram of plant DNA (pg ng^−^^1^ plant DNA).

### 2.6 Genome‐wide association studies

Genome‐wide association analysis was conducted on the 190 genotypes using phenotypic mycotoxin data and 4881 high‐quality SNPs provided by the Rocinante Lab (Isidro‐Sánchez et al., 2020). A fixed‐effect model was fitted to obtain BLUE values using:

$$y_{\mathrm{ij}}=\mu +gi+t\left(s\right)j+\varepsilon$$

where *y_ij_* is the observed value on the *i*th individual and *j*th trial, *t*(*s*)*_j_* is the nested effect of samples within trials, and *ϵ* is the residual having a distribution with a mean of zero and a variance of *σ* ^2^. SNP‐trait associations were evaluated using the GAPIT package (Wang & Zhang, 2021) and the BLINK algorithm (Huang et al., 2019). Significance thresholds for SNP markers were determined using Bonferroni correction and false discovery rate control (FDR) (Benjamini & Hochberg, 1995).

The Mrg designations used here refer to merged linkage groups from a genotyping‐by‐sequencing (GBS) consensus map built by combining many biparental oat populations (Chaffin et al., 2016). Because hexaploid oat (2*n* = 6*x* = 42; A, C, and D genomes) has undergone inter‐genomic translocations, a single consensus group can contain markers that, in a physically assembled genome, belong to more than one chromosome. Mrg11 is one such group; different segments correspond to chromosomes 1C, 4C, and 6A in the OT3098 v2 assembly. Consequently, two markers sharing the label Mrg11 are not necessarily physically linked, and QTL reported on Mrg11 in different studies may lie on different chromosomes. Throughout this paper, we therefore report physical chromosome positions (OT3098 v2) as the primary coordinate and retain the consensus cM position only to indicate marker provenance. Physical positions were obtained by anchoring each GBS tag to the reference assembly; where a tag matched more than one position (a duplicated or homoeologous tag), all matches are reported (Table 1).

**TABLE 1:** Significant single‐nucleotide polymorphisms (SNPs) associated with T‐2 and HT‐2 toxin accumulation, showing genome‐wide association study (GWAS) positions and mapped physical coordinates in the OT3098 v2 genome.

SNP marker	LG	Position (cM)	*p*‐value	MAF	*β* (effect)	Chr	Position (bp)
avgbs_cluster_11885.1.13	Mrg11	3.7	3.40 × 10^−^^6^	0.021	−108.9	6A	432,536,037
avgbs_cluster_11885.1.22	Mrg11	3.7	3.40 × 10^−^^6^	0.021	−108.9	6A	432,536,037
avgbs_cluster_11885.1.28	Mrg11	3.7	3.40 × 10^−^^6^	0.021	−108.9	6A	432,536,037
avgbs_cluster_13857.1.17	Mrg11	9.8	1.22 × 10^−^^5^	0.047	−69.4	4C	21,540,477
avgbs_cluster_39187.1.31	Mrg11	9.8	1.22 × 10^−^^5^	0.047	−69.4	4C	21,549,732

Note: Markers avgbs_cluster_11885.1.13, avgbs_cluster_11885.1.22, and avgbs_cluster_11885.1.28 mapped to the same physical position on chromosome 6A and therefore represent a single SNP locus. Negative effect (*β*) values indicate alleles associated with reduced T‐2 and HT‐2 toxin accumulation.

Abbreviations: bp, base pairs; Chr, chromosome; cM, centimorgan; LG, linkage group; MAF, minor allele frequency; β, estimated SNP effect.

### 2.7 Identification of candidate genes associated with significant SNPs

Significant SNPs from the GWAS were mapped to physical genomic coordinates using the T3/Oat genome browser (https://oat.triticeaetoolbox.org), which reports positions relative to the *Avena sativa* OT3098 v2 reference genome (Morales et al., 2022). For each SNP, the corresponding chromosome and base‐pair position was recorded. Three markers from the avgbs_cluster_11885 group mapped to the same physical position on chromosome 6A (432,536,037 bp) and were therefore treated as a single locus in downstream analyses.

Annotated gene models were obtained from the PepsiCo_OT3098_V2_panoat_nomenclature.gff3 file (downloaded from GrainGenes/T3). SNP‐centered windows of ± 30 kb were constructed around each associated locus, and overlapping gene models were identified using *GenomicRanges* (Lawrence et al., 2013). Gene models located within these windows were extracted together with their available functional annotations.

For the chr6A candidate gene (HIPP39), the predicted protein sequence was analyzed using BLASTP (Altschul, 1997) against the NCBI non‐redundant protein database, restricted to *Poaceae* (taxid: 4479), including *Avena sativa*, *Triticum aestivum* L., and *Hordeum vulgare* L., to identify homologous proteins. Functional annotation was further assessed using UniProtKB (The UniProt Consortium, 2023) and the Barlex barley genome database (Colmsee et al., 2015). Gene ontology terms were retrieved to infer molecular function and biological processes (Ashburner et al., 2000). Conserved domains were identified using UniProt (InterPro/Pfam annotations) (Blum et al., 2021; Mistry et al., 2021).

### 2.8 Statistical analysis

Statistical analyses were performed in R (RStudio 2025.09.2). For field trials, plot‐level means (*n* = 3 per genotype per site) were used for all comparisons. For glasshouse experiments, each genotype was evaluated across two independent trials with two replicate measurements per trial (four observations per genotype).

Toxin values below the assay detection limit (20 µg kg^−^^1^) were assigned the detection limit for analysis. Because toxin data were non‐normally distributed (Shapiro–Wilk, *p* < 0.001), log‐transformed values [log (Toxin + 1)] were used for parametric analysis. For the glasshouse dataset, a linear model was fitted to log‐transformed toxin values to assess the effects of genotype and trial. Model assumptions were evaluated by inspection of residual plots, Shapiro–Wilk tests for normality, and Levene's test for homogeneity of variance.

For field trials, genotype effects within each site were assessed using the Kruskal–Wallis test, with effect size reported as epsilon‐squared (*ε* ^2^). When significant, pairwise comparisons among genotypes were performed using Dunn's test with Benjamini–Hochberg correction. Differences between field sites were evaluated using paired Wilcoxon signed‐rank tests applied to genotype means. Associations between fungal DNA and toxin concentrations were quantified using Spearman's rank correlation (*ρ*), as the data were not normally distributed. All visualizations were generated using the ggplot2 package.

## 3 RESULTS

### 3.1 Field evaluation of oat genotype susceptibility to *F. langsethiae* and associated toxin accumulation

Nineteen oat genotypes were evaluated for the presence of toxigenic *F. sporotrichoides* and *F. langsethiae* (qPCR) and T‐2 + HT‐2 toxin accumulation (ELISA) under field infection conditions at two sites in 2022 (UCD Lyons and Teagasc Oak Park). *F. sporotrichioides* was not detected in any oat sample. *F. langsethiae* DNA levels were substantially higher in oats from the Teagasc site compared with UCD Lyons (paired Wilcoxon test, *p* = 0.0005) (Figure 1). Genotype differences were significant at both sites, with stronger effects observed at Teagasc (*p* < 0.001, *ε* ^2^ = 0.48) than at UCD Lyons (*p* = 0.024, *ε* ^2^ = 0.24). Among the genotypes, Elen, Blythe, and 021‐S02011Au16 consistently exhibited the highest *F. langsethiae* DNA concentrations (1.55–1.70 pg ng^−^^1^), whereas Sandy, Conway, and Milo displayed the lowest colonization levels (0.26–0.33 pg ng^−^^1^). Jokikyla ME0501 showed exceptionally low infection at UCD Lyons, with significantly lower infection compared to the most susceptible genotype, Stormont Arrow (*p* = 0.032). Despite location‐specific differences in infection intensity, genotype performance was broadly consistent (Figure 1), indicating relatively stable genotype‐dependent susceptibility patterns to *F. langsethiae*.

**FIGURE 1:**
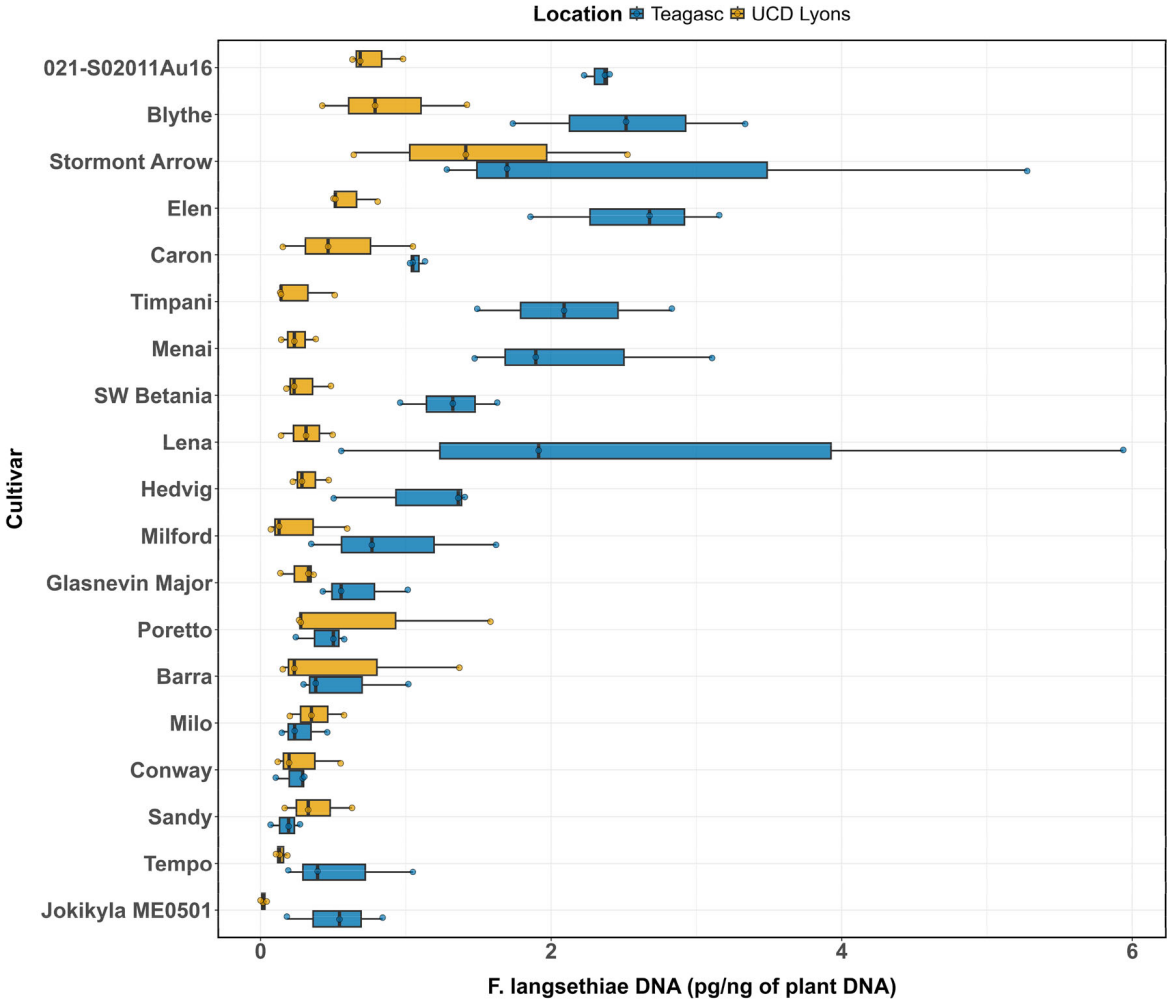
Quantification of *Fusarium langsethiae* DNA in 19 oat genotypes at two Irish field sites in 2022 (Teagasc and UCD Lyons). Fungal DNA was quantified by quantitative polymerase chain reaction (qPCR) and expressed as pg *F. langsethiae* DNA per ng plant DNA. Boxplots show medians, interquartile ranges, and points for independent biological replicates (*n* = 3 per genotype per site). Each biological replicate was analyzed using three technical qPCR replicates, which were averaged prior to statistical analysis. Overall, DNA levels were higher at Teagasc than at the UCD Lyons site (paired Wilcoxon, *p* = 0.0005).

T‐2 and HT‐2 toxin concentrations ranged from 20 to 562 µg kg^−^^1^ across environments, with comparable mean values at the two sites (UCD Lyons: 169 ± 144 µg kg^−^^1^; Teagasc: 156 ± 114 µg kg^−^^1^) and no significant location effect (*p* = 0.595) (Figure 2). Genotype effects on toxin accumulation were significant at both sites, although substantially stronger at Teagasc (*p* < 0.001, *ε* ^2^ = 0.523) than at UCD Lyons (*p* = 0.0296, *ε* ^2^ = 0.23). Blythe consistently exhibited elevated toxin accumulation across both locations (171–443 µg kg^−^^1^), whereas Tempo, Milo, and Glasnevin Major maintained the lowest levels (53–120 µg kg^−^^1^). Post hoc comparisons (Dunn's test with Benjamini–Hochberg correction) were significant only at Teagasc, where Blythe accumulated higher toxin concentrations than Glasnevin Major (padj = 0.039), Tempo (padj = 0.042), and Milo (padj = 0.047). At UCD Lyons, although the overall genotype effect was significant, pairwise comparisons were not.

**FIGURE 2:**
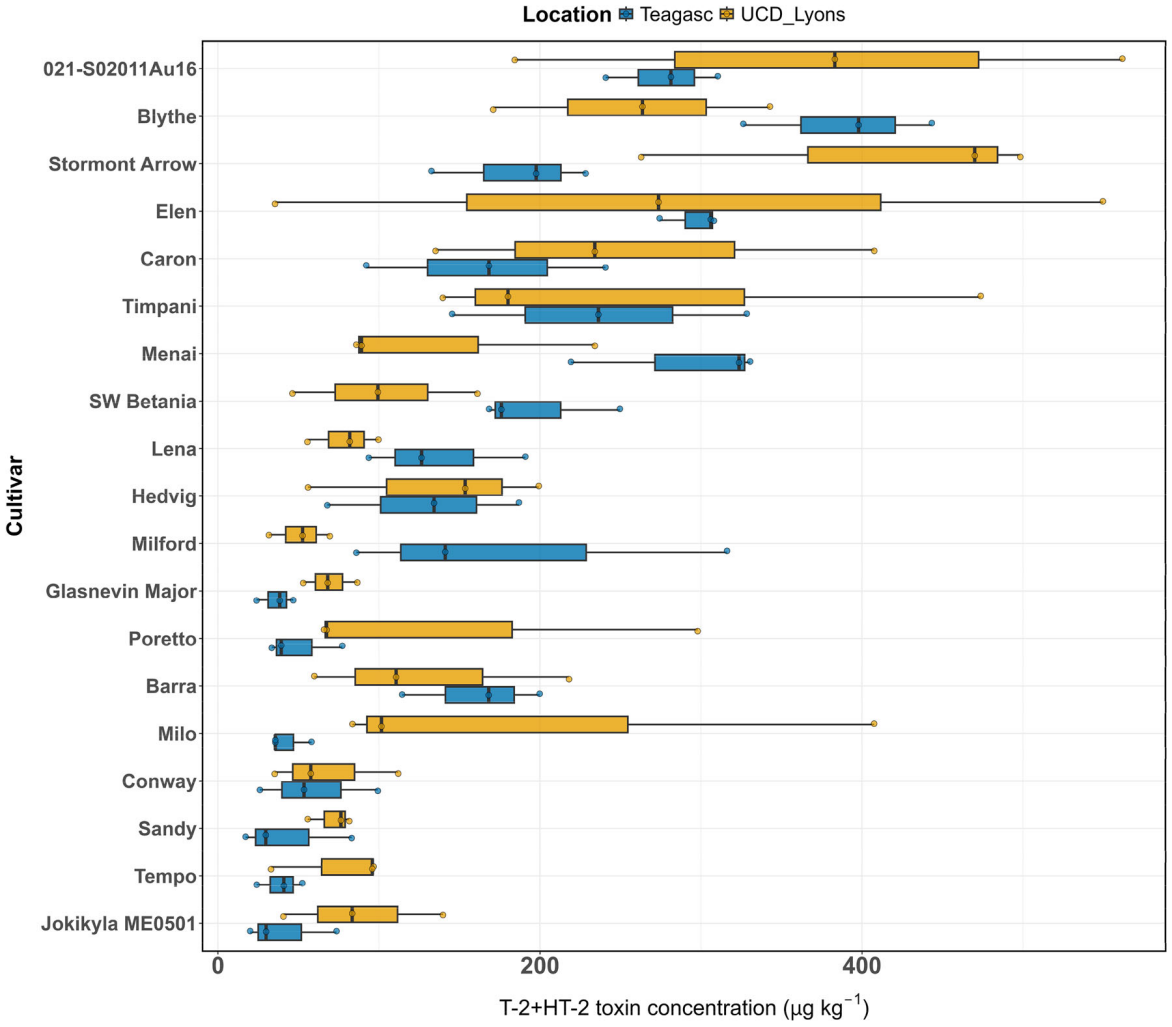
T‐2 and HT‐2 toxin concentrations in 19 oat genotypes grown at two field sites in 2022 (Teagasc and UCD Lyons). Toxin levels were quantified using enzyme‐linked immunosorbent assay (ELISA). Boxplots show median values with interquartile ranges, and individual points represent independent biological replicates (*n* = 3 per genotype per location). Each biological replicate was analyzed using two technical ELISA replicates, which were averaged prior to statistical analysis. Kruskal–Wallis tests indicated significant genotype effects at both locations (Teagasc: *p* < 0.001; UCD Lyons: *p* = 0.030).

Correlation analysis revealed a strong positive association between *F. langsethiae* DNA content and toxin accumulation (Figure 3). Spearman's correlation was significant overall (*ρ* = 0.62, *p* < 0.001) and remained consistent across both locations (Teagasc: *ρ* = 0.74, *p* < 0.001; UCD Lyons: *ρ* = 0.63, *p* = 0.004). Analysis of individual biological plot data (*n* = 114) showed a similarly strong positive correlation (*ρ* = 0.59, *p* < 0.001), indicating that the association was robust and independent of averaging biological replicates. This trend was reflected in genotype performance: five genotypes displayed consistently high susceptibility, with concurrently elevated *F. langsethiae* DNA and T2 + HT2 toxin levels: Stormont Arrow (DNA = 2.140 pg ng^−^^1^; Toxins = 298.4 µg kg^−^^1^), Blythe (1.704; 324.2), Elen (1.586; 291.2), 021‐S02011Au16 (1.550; 327.0), and Menai (1.205; 213.8). In contrast, five genotypes exhibited strong resistance, characterized by consistently low fungal colonization and toxin accumulation: Glasnevin Major (DNA = 0.471 pg ng^−^^1^; Toxins = 52.8 µg kg^−^^1^), Tempo (0.342; 57.2), Sandy (0.276; 57.3), Jokikyla ME0501 (0.271; 64.6), and Conway (0.260; 64.0).

**FIGURE 3:**
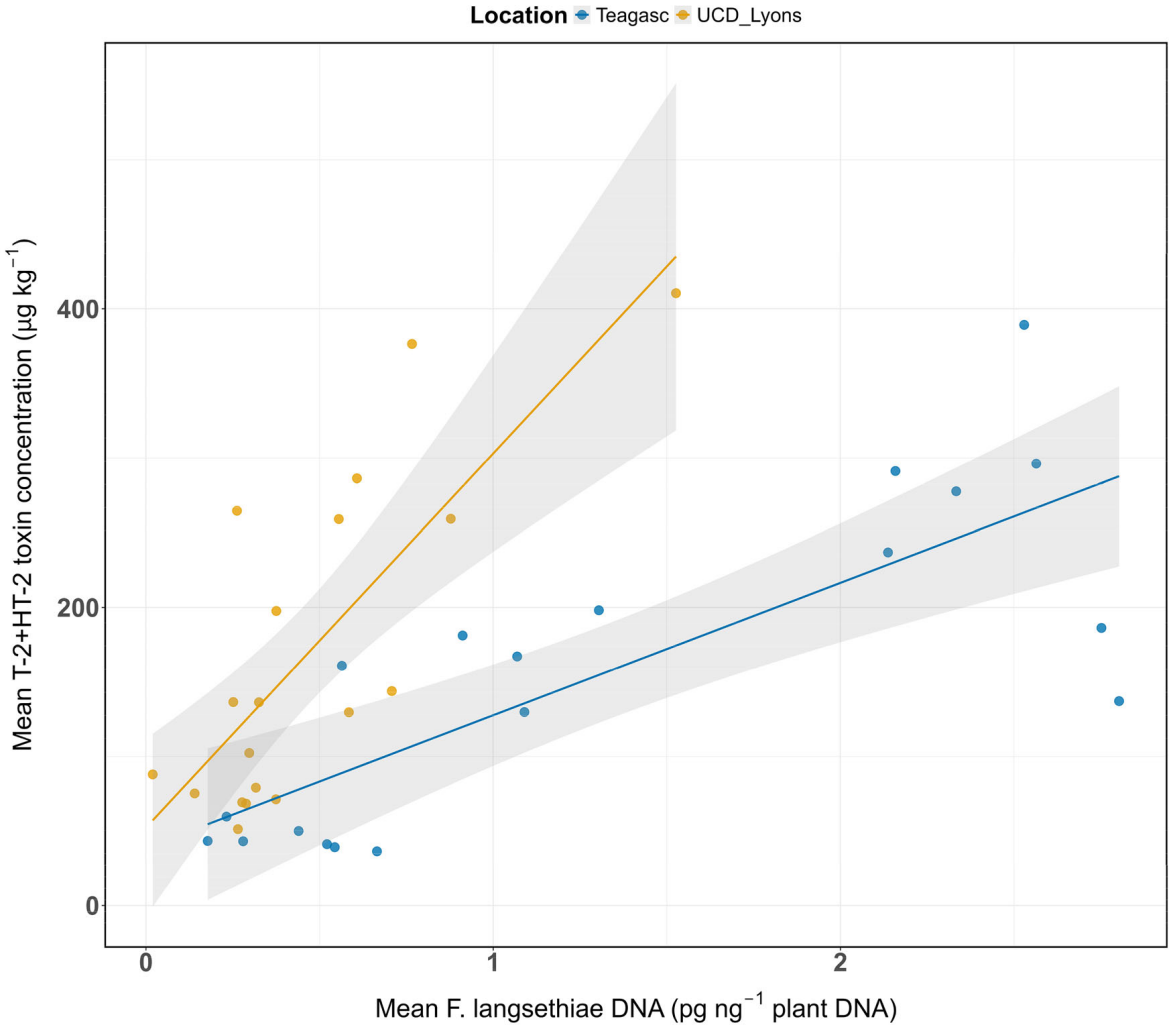
Relationship between *Fusarium langsethiae* DNA colonization and T‐2 + HT‐2 toxin accumulation across two field sites. Each point represents the mean for one genotype at one location (*n* = 3 plots per mean). Colors distinguish Teagasc and UCD Lyons. Lines show simple linear regression fits with 95% confidence bands for each location. Spearman rank correlations between fungal DNA (pg ng^−^^1^ plant DNA) and toxin concentration (µg kg^−^^1^) were significant overall (*ρ* = 0.62, *p* < 0.001) and within both Teagasc (*ρ* = 0.74, *p* < 0.001) and UCD Lyons (*ρ* = 0.63, *p* = 0.004).

### 3.2 Comparative performance of genotypes under field and glasshouse environments

Of the 19 genotypes assessed under field conditions, 11 were also evaluated under glasshouse conditions for T‐2 and HT‐2 toxin accumulation in *F. langsethiae*‐inoculated plants as part of the larger study of 190 genotypes (Figure 4). The overall relationship between field and glasshouse mean toxin concentrations was weak and not statistically significant (Spearman's *ρ* = 0.17, *p* = 0.61; Figure S1). However, a subset of genotypes showed consistent toxin accumulation in both the glasshouse and the field environments. For instance, Tempo (field: 57.2 µg kg^−^^1^; glasshouse: 97.4 µg kg^−^^1^), Sandy (field: 57.3 µg kg^−^^1^; glasshouse: 97.4 µg kg^−^^1^) and Jokikyla ME0501 (field: 64.6 µg kg^−^^1^; glasshouse: 61.9 µg kg^−^^1^) were the lowest toxin‐accumulating genotypes in both environments. In contrast, Stormont Arrow displayed consistently high toxin concentrations (field: 298 µg kg^−^^1^; glasshouse: 149 µg kg^−^^1^), indicating a stable susceptible phenotype.

**FIGURE 4:**
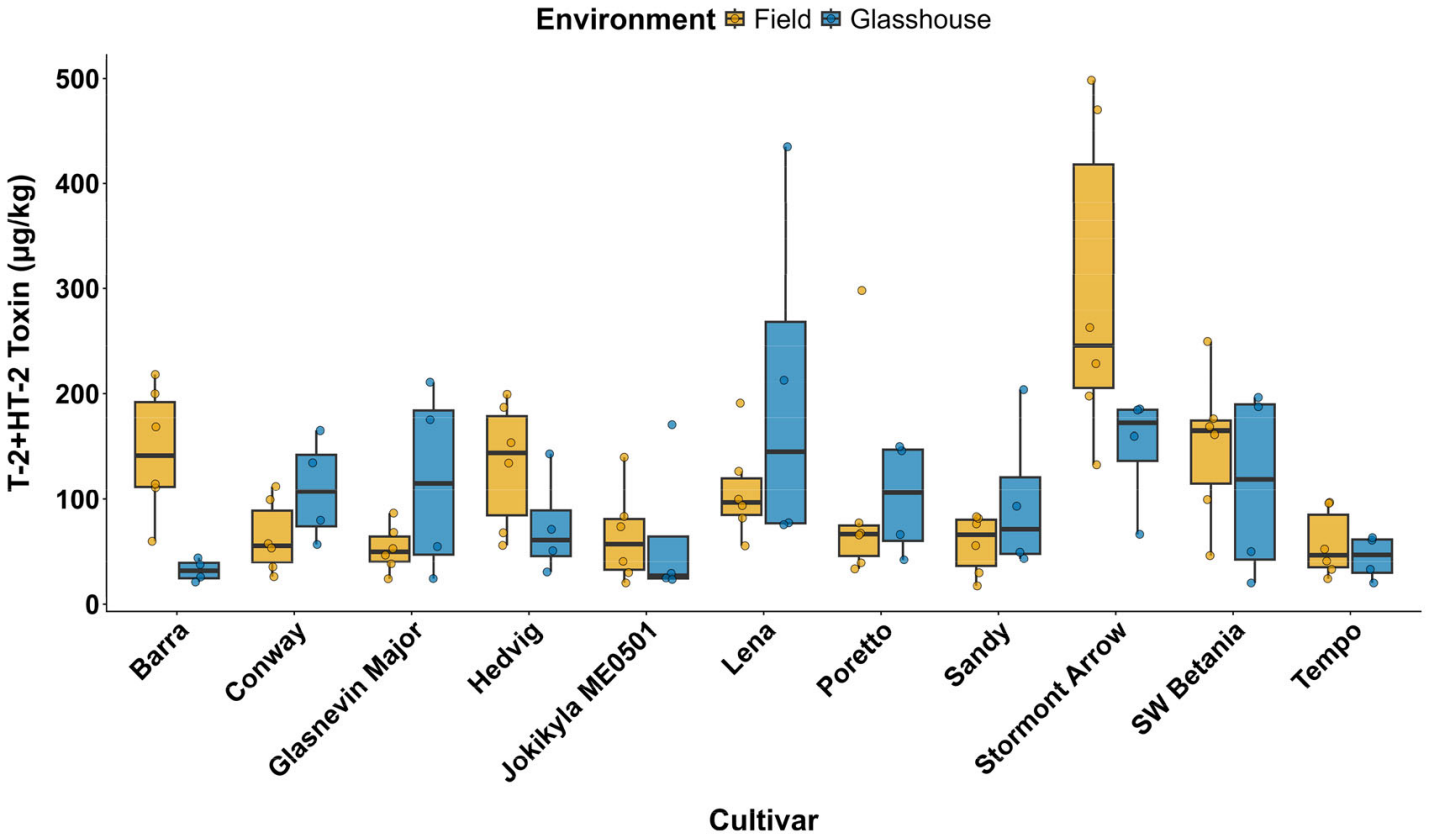
Comparison of T‐2 and HT‐2 toxin accumulation in 11 oat genotypes across both field conditions (natural inoculum) and glasshouse studies (*Fusarium langsethiae* inoculum). Boxplots represent the distribution of toxin concentrations for each genotype, with points representing individual replicates (Field: *n* = 6; Glasshouse: *n* = 4).

Despite these similarities, we also observed that several genotypes showed greater differences in toxin accumulation across environments, demonstrating the influence of the infection system and environmental conditions. Particularly, Barra showed higher toxin levels under natural field infection (145 µg kg^−^^1^) than under controlled inoculation (32.1 µg kg^−^^1^). In contrast, Glasnevin Major exhibited relatively low field toxin levels (52.8 µg kg^−^^1^) but higher accumulation in the glasshouse (116.3 µg kg^−^^1^).

### 3.3 Glasshouse evaluation of toxin accumulation across 190 oat genotypes

A panel of 190 oat genotypes was evaluated for T‐2 and HT‐2 accumulation in response to *F. langsethiae* inoculation under controlled glasshouse conditions. Each genotype was assessed across two independent trials with two replicate measurements per trial (four observations per genotype; Table S5). Toxin levels exhibited substantial genotypic variability, ranging from the assay detection limit (20 µg kg^−^^1^) to 1000 µg kg^−^^1^, with a strongly right‐skewed distribution (Figure 5A). The median concentration was 50.1 µg kg^−^^1^ and the mean was 122.7 ± 172.6 µg kg^−^^1^, indicating that most genotypes accumulated low toxin levels while a subset showed high susceptibility. Mock‐inoculated control plants consistently contained T‐2 and HT‐2 toxin concentrations below the assay detection limit (≤20 µg kg^−^^1^), indicating the absence of detectable background contamination in the glasshouse experiments.

**FIGURE 5:**
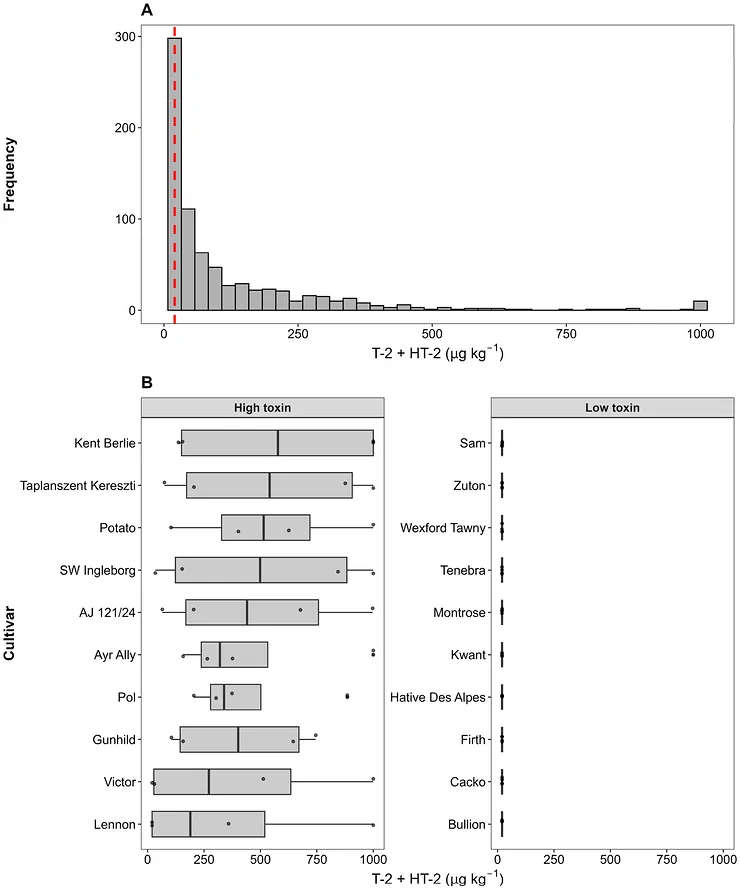
Distribution and genotype‐specific variation of T‐2 + HT‐2 toxin accumulation in *Fusarium langsethiae*‐inoculated oat genotypes under glasshouse conditions. (A) Histogram showing the right‐skewed distribution of toxin concentrations (µg kg^−^^1^) across all observations from 190 genotypes evaluated in two independent trials with two replicate measurements per trial (*n* = 760 total observations; 4 per genotype). The red dashed line indicates the enzyme‐linked immunosorbent assay (ELISA) detection limit (20 µg kg^−^^1^). (B) Boxplots showing replicate‐level variation in the 10 most and least susceptible genotypes, ranked by mean toxin concentration. Each boxplot is based on four observations per genotype. Boxes represent interquartile ranges, horizontal lines indicate medians, whiskers show the range, and points represent individual observations. Genotypes with values at the detection limit represent no detectable toxin accumulation and cannot be distinguished below 20 µg kg^−^^1^.

A linear model fitted to log‐transformed toxin values revealed significant effects of both trial (*F* = 58.46; df = 1, 569; *p* < 0.001) and genotype (*F* = 2.44; df = 189, 569; *p* < 0.001), indicating that toxin accumulation varied significantly among genotypes and between experimental runs. Genotype toxin means were weakly correlated between runs (Pearson *r* = 0.02; Spearman *ρ* = 0.16). This between‐run instability is reflected in the wide replicate spread evident in Figure 5B, where measurements for an individual genotype ranged from the assay detection limit to nearly 1000 µg kg^−^^1^. The ten genotypes with the highest and lowest overall mean toxin concentrations are shown in Figure 5B. The most susceptible genotypes included Kent Berlie, Taplanszent Kereszti, Potato, SW Ingleborg, AJ 121/24, Ayr Ally, Pol, Gunhild, Victor, and Lennon, with mean toxin concentrations ranging from 350 to 573 µg kg^−^^1^. In contrast, nine genotypes (Bullion, Cacko, Firth, Hative des Alpes, Kwant, Montrose, Tenebra, Wexford Tawny, and Zuton) showed toxin concentrations consistently at the assay detection limit (20 µg kg^−^^1^) across all observations, indicating no detectable toxin accumulation under the conditions tested. Sam also showed very low toxin levels close to the detection limit.

### 3.4 Genome‐wide association analysis

A total of 4881 SNP markers were evaluated for association with T‐2 and HT‐2 toxin accumulation using the BLINK model in the GAPIT package, with FDR correction applied (Table S6). Five SNPs met the significance threshold, and three of these surpassed the Bonferroni cutoff (Table 1; Figure 6). All significant markers fall on a single GBS consensus linkage group (Mrg11) but map to two physically distinct regions of the OT3098 v2 assembly, chromosomes 6A and 4C (Table 1), at consensus positions 3.7 cM and 9.8 cM. All markers had low minor allele frequencies (2.1%–5.1%). At 3.7 cM, three SNPs exhibited strong association signals, each with *p* = 3.40 × 10^−^^6^. A second cluster of two SNPs at 9.8 cM also showed significant associations (*p* = 1.22 × 10^−^^5^). These results identify two physically distinct loci (chromosomes 6A and 4C, OT3098 v2) associated with variation in T‐2 and HT‐2 toxin accumulation among the tested oat genotypes. Because the three markers at 3.7 cM correspond to a duplicated GBS tag that maps equally well to chromosomes 6A and 4C, the 6A assignment reflects the position of the candidate gene identified below (Section 3.5). When the two glasshouse runs were analyzed separately, these associations were recovered in only one run (Supporting Information S7), consistent with the weak between‐run correlation of toxin phenotypes (Pearson *r* = 0.02) and reinforcing the need for independent validation.

**FIGURE 6:**
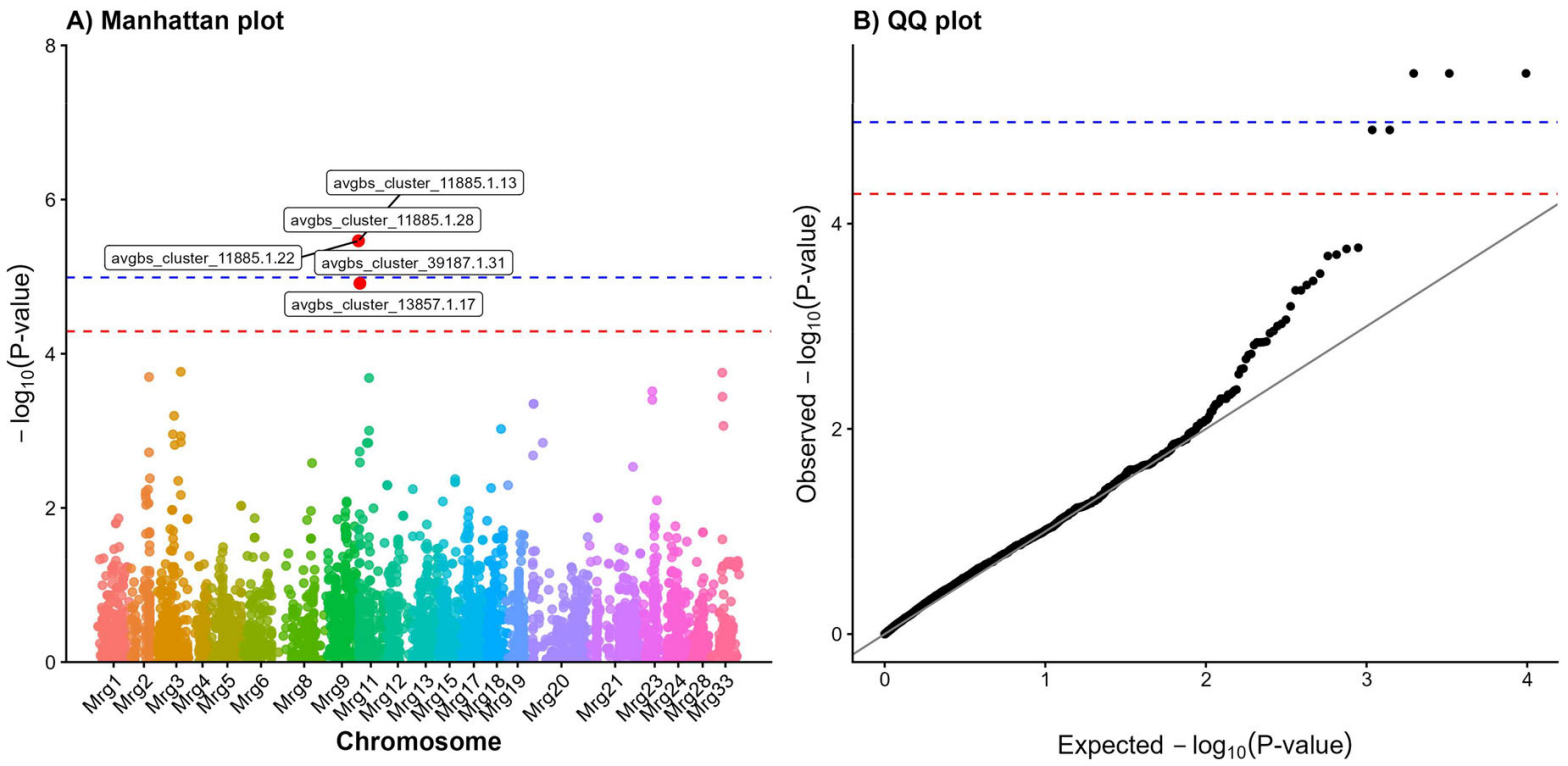
Manhattan plot and quantile–quantile (*Q*–*Q*) plot from the genome‐wide association study (GWAS) for the given mycotoxin T‐2 + HT‐2 trait. (A) Manhattan plot with labels for the significant single‐nucleotide polymorphisms (SNPs) above red dashed line (FDR) and the blue dashed line (Bonferroni); (B) *Q*–*Q* plot with threshold for FDR (red dashed line) and Bonferroni (blue dashed line).

### 3.5 Identification of candidate genes associated with significant SNPs

The five significant SNP markers identified in the GWAS were mapped to the OT3098 v2 reference genome. Three markers from the *avgbs_cluster_11885* group all mapped to the same physical position on chromosome 6A (432,536,037 bp), confirming that they represent a single underlying SNP. The remaining two markers mapped to distinct positions on chromosome 4C (21,540,477 bp and 21,549,732 bp) (Table 1).

To identify candidate genes associated with these loci, ± 30 kb windows were examined around each SNP. At the chr6A locus, only one annotated gene was present within this interval: AVESA.00001b.r3.6Ag0003188, encoding HIPP39 (Heavy Metal Associated Isoprenylated Plant Protein 39). This gene lies approximately 450 bp from the SNP and was the only annotated protein‐coding gene within the window. HIPP proteins are involved in metal binding, redox regulation, and stress‐responsive signaling (De Abreu‐Neto et al., 2013), making HIPP39 a strong positional candidate. BLASTP analysis identified homologous proteins across grasses, including *Triticum aestivum* and related species, with sequence identity ranging from 74% to 90% and query coverage of 59%–72% (Table S8). These homologs were consistently annotated as heavy metal‐associated isoprenylated plant proteins (HIPP family). Functional annotation using UniProtKB and the Barlex barley genome database supported this classification (Table S9). Domain analysis further confirmed the presence of a conserved heavy metal‐associated domain (results not shown), a defining feature of HIPP proteins. Together, these results support HIPP39 as a high‐confidence candidate gene at the chr6A locus, with a putative role in metal ion binding and stress‐related processes.

In contrast, no annotated protein‐coding genes were detected within ± 30 kb of either SNP on chromosome 4C. The closest annotated genes, an F‐box protein and a class III peroxidase, were located 105–115 kb from the SNP positions, outside the search window. These loci may therefore lie within intergenic or regulatory regions not represented in the current OT3098 v2 genome annotation.

## 4 DISCUSSION

In our Irish field trials, we observed substantial variation in T‐2 and HT‐2 toxin concentrations in oats from across two field sites. All genotypes remained below the European Commission threshold (1250 µg kg^−^^1^) for unprocessed oats (European Commission, 2024b). The toxin levels observed at both Teagasc and UCD Lyons field sites were in line with previous European surveys, such as those from Sweden and the United Kingdom, which have also documented notable T‐2 and HT‐2 toxin occurrence in oats under natural conditions (Edwards & Stancic, 2024; Fredlund et al., 2013). We detected *F. langsethiae* DNA in all genotypes across both field sites, which confirms its prevalent occurrence in European oats (De Colli et al., 2021; Edwards et al., 2012; Fredlund et al., 2010; Kolawole et al., 2021). In agreement with previous studies (Fredlund et al., 2013; Schnerr et al., 2002; Waalwijk et al., 2004), fungal DNA levels exhibited a strong correlation with toxin accumulation (*ρ* = 0.62, *p* < 0.001). In contrast, *F. sporotrichioides* was undetectable in our samples, consistent with UK reports in which this species was rarely identified despite high T‐2 and HT‐2 levels (Bucur et al., 2025; Edwards et al., 2012; Natarajan et al., 2025). A recent nationwide survey of commercial oats found that *F. langsethiae* was the most prevalent species, detected in 94% of fields. In contrast, species such as *F. tricinctum*, *F. poae*, *F. avenaceum*, and *F. culmorum* were also isolated, typically at lower frequencies and relative abundance (Bucur et al., 2025). This supports the conclusion that, despite the presence of multiple *Fusarium* species in field‐grown oat panicles, *F. langsethiae* remains the dominant contributor to T‐2 and HT‐2 contamination in Irish oats. Because the field trials relied on natural infection, variation in local inoculum pressure and microenvironmental conditions was expected and likely contributed to the biological variability observed among replicate plots (Oerke et al., 2010). A limitation of the present study is that field plot coordinates were unavailable for spatial analysis, preventing assessment of whether localized hotspots of infection contributed to the observed variability among biological replicates.

Despite fundamental differences in infection methodology (natural vs. artificial) and environmental conditions (field vs. glasshouse), a small number of the 11 genotypes common to our field and glasshouse study, including Tempo, Sandy, and Jokikyla ME0501, consistently showed low toxin accumulation in both environments, whereas Stormont Arrow remained highly susceptible. Similarly, a subset of cultivars consistently exhibited either low or high T‐2 and HT‐2 toxin accumulation in both our glasshouse study and the field study previously conducted by Isidro‐Sánchez et al. (2020) (low = Sandy, Tempo, Jokikyla ME0501, Conway, and Glasnevin Major; high = Gniady, Triumph, Furman, Stormont Iris, Dippawski, Gunhild, and Potato). These results indicate that although the environment strongly influences absolute toxin accumulation, stable resistance and susceptibility phenotypes can still be identified across contrasting environmental and experimental conditions (Edwards & Stancic, 2024; Hofgaard et al., 2022).

Despite artificial inoculation at flowering, toxin levels in the glasshouse were generally lower than those observed under field conditions, a discrepancy previously reported (Aamot et al., 2022; Martin et al., 2018). A similar discrepancy has been reported previously in oat, where Imathiu (2008) observed that glasshouse inoculation experiments with *F. langsethiae* failed to reach the high HT‐2 and T‐2 concentrations commonly found in commercial oat fields. Differences are likely attributable to environmental differences, as post‐inoculation humidity and temperature strongly regulate *F. langsethiae* colonization and mycotoxin production (Aamot et al., 2022; Nazari et al., 2016). Plants grown in a glasshouse are typically exposed to shorter periods of high humidity and more regulated temperatures than in the field, which may limit toxin accumulation compared with field conditions. Several non‐mutually exclusive factors likely contribute to this discordance. First, only 11 genotypes were common to both systems, limiting the strength of the field–glasshouse comparison (Spearman *ρ* = 0.17, *p* = 0.61). Second, the two systems assess different components of resistance. Natural field infection integrates FHB disease escape and avoidance traits, such as flowering time, plant height, and panicle architecture, which determine the extent of inoculum exposure and panicle colonization (Buerstmayr et al., 2009; Mesterházy, 2020). In contrast, direct spray inoculation at anthesis largely bypasses these traits and primarily assesses post‐infection physiological resistance. Consequently, genotypes differing in phenology may escape peak inoculum pressure or favorable environmental conditions in the field while being fully exposed under artificial inoculation. Third, field infection depends on heterogeneous, uncontrolled, and potentially multi‐species inoculum, together with post‐anthesis humidity and temperature, which are major drivers of pathogen colonization and mycotoxin production. The approximately twofold difference in *F. langsethiae* DNA observed between the two field sites further illustrates the strong influence of environmental conditions on field infection. Agronomic traits were recorded at both field sites, and differences between sites were observed, particularly in disease pressure, lodging, and yield. However, as these agronomic data form part of a separate study, their relationships with *F. langsethiae* colonization and toxin accumulation were not evaluated here. Consequently, their potential contribution to variation in toxin accumulation and genotype × environment interactions could not be assessed directly. Agronomic traits have previously been shown to influence mycotoxin contamination in other cereal pathosystems (Battilani et al., 2008). Future studies integrating agronomic traits with environmental variables and toxin accumulation will be important for improving our understanding of genotype × environment interactions.

Overall, we found no significant correlation in the toxin levels detected for the 11 varieties commonly tested in both the field and the glasshouse; nor was there any correlation between the results previously obtained for the 190 genotypes in the field and our glasshouse results (results not shown). Hautsalo et al. (2020) analyzed 406 genotypes and similarly reported no clear relationship and inconsistent susceptibility rankings for FHB and DON between greenhouse and field environments. As in our study with Barra and Glasnevin Major, Hautsalo et al. (2020) also found that the variety BOR31 was highly susceptible in the greenhouse but only moderately susceptible in the field. Beyond environmental factors, plant architectural traits can also contribute to FHB inconsistency (Hautsalo et al., 2020; He et al., 2013). For example, hull trichome density has recently been identified as a factor influencing FHB susceptibility in oats, as these microstructures facilitate spore attachment and colonization (Schurack et al., 2025). However, because these passive traits are also highly sensitive to environmental conditions, this leads to significant inconsistencies in genotype rankings observed across diverse environments.

GWAS analysis identified five SNPs significantly associated with reduced T‐2 and HT‐2 toxin accumulation under controlled glasshouse conditions. The negative SNP effects observed in this study suggest that allelic variation at this locus contributes to reduced toxin accumulation. These markers define two physically distinct loci, on chromosomes 6A and 4C of the OT3098 v2 assembly, that share a single GBS consensus linkage group (Mrg11). They therefore represent a genetic architecture distinct from the Mrg06 (field) QTL previously detected in this population (Isidro‐Sánchez et al., 2020). Bjørnstad et al. (2017) also detected a DON and FHB‐associated QTL on Mrg11/1C in oats under *F. graminearum* field infection. However, the Bjørnstad locus maps to the chromosome 1C segment of Mrg11, whereas the loci reported here map to the 6A and 4C segments. Because a single consensus (Mrg) group can span more than one physical chromosome (Section 2.6), shared Mrg11 membership does not indicate physical co‐location; these are different loci that merely carry the same consensus‐group label. Rather than a single genomic region being repeatedly implicated in *Fusarium* toxins across different experimental systems, the recurrence of the Mrg11 label across studies instead reflects the composite, multi‐chromosome nature of this consensus group. In cereals, FHB resistance is a composite trait whose phenotypic expression is strongly modulated by environment (Ma et al., 2022; Mesterhazy, 2020). Toxin‐related resistance components (commonly referred to as Type IV or V) are particularly sensitive to inoculation method, pathogen isolate, and climatic conditions during infection. The detection of these associations under controlled glasshouse conditions, but their inconsistent recovery across independent glasshouse trials and previous field experiments, suggests that their expression may be sensitive to environmental and experimental variation. In contrast, field environments are characterized by variable infection pressure, fluctuating weather conditions, and the co‐occurrence of multiple *Fusarium* species alongside diverse microbial taxa, all of which can mask genetic effects and reduce QTL detectability. Such variability in QTL detection most likely reflects genotype‐by‐environment interactions governing toxin accumulation rather than a fundamentally different resistance mechanism (Buerstmayr et al., 2009; Mesterhazy, 2020). These findings indicate that marker‐trait associations identified in a single environment should be interpreted cautiously and require validation across multiple environments and years before they can be reliably applied in marker‐assisted breeding. Future validation using the complete PanOat pangenome graph will help confirm the physical positions of these loci across diverse oat genomes and refine candidate gene identification (Avni et al., 2025). Similar QTL instability related to FHB and associated toxins across environments has been reported in oats. For instance, He et al. (2013) and Bjørnstad et al. (2017) showed that several resistance loci were not consistently detected across years, partly due to environmental variation and phenology‐related disease escape. Similar patterns are also well documented in wheat‐FHB studies (Löffler et al., 2009; Zheng et al., 2021). For instance, Haile et al. (2023) identified 31 QTLs for FHB resistance in durum wheat, but only nine were consistent across environments.

We examined the genomic regions nearby significant SNPs. Candidate gene analysis identified a single annotated gene, HIPP39, within a ± 30 kb window at the chromosome 6A locus. Sequence similarity analysis and functional annotation confirmed that this gene belongs to the HIPP family. HIPP proteins constitute a metallochaperone‐like protein family that has been implicated in metal detoxification, redox homeostasis, and stress‐responsive signaling mechanisms (Barr et al., 2023; De Abreu‐Neto et al., 2013; Zhang et al., 2020). Several cereal HIPP genes exhibited induction during pathogen attack, and constituents of this family have emerged as critical host targets for fungal effectors. For example, the *Magnaporthe oryzae* effector Pwl2 has been shown to interact with barley HIPP43, leading to its displacement from plasmodesmata and subsequent inhibition of PAMP (pathogen‐associated molecular patterns)‐triggered immunity (PTI) and increased disease susceptibility (Were et al., 2025). Similarly, an effector of *Rhizoctonia solani* has also been reported to bind rice HIPPs, leading to the modification of host immune‐associated responses (Niu et al., 2023). Taken together, these findings indicate that HIPP proteins are evolutionarily conserved host factors implicated in immune regulation and are frequently targeted by pathogens. The association of the chr6A locus containing HIPP39 with variation in T‐2 and HT‐2 accumulation, therefore, highlights a genomic region harboring a biologically relevant host target. Genetic variation or differences in gene regulation in this region may influence the interaction between oat plants and *F. langsethiae*. However, this study does not address the direct causal relationship of HIPP39, and additional functional validation studies would be required to determine whether this gene has any role in toxin resistance or susceptibility. The evolutionary relationship between oats and *F. langsethiae* remains poorly understood, and loci such as HIPP39 that are associated with toxin accumulation and previously found to encode effector targets provide a potential entry point for examining whether host–pathogen co‐evolution has shaped toxin‐related traits in oats.

In contrast, none of the SNPs mapped to chromosome 4C co‐localized with annotated coding genes within ± 30 kb, suggesting that this region may contain regulatory elements not yet captured in the OT3098 v2 assembly. The contrasting contexts imply that chr6A and chr4C influence toxin‐related outcomes via distinct mechanisms, with HIPP39 as a strong positional candidate, while the chr4C region may involve regulatory variation contributing to reduced toxin accumulation.

This study identified oat genotypes that consistently accumulated low levels of *F. langsethiae* DNA and T‐2 + HT‐2 toxins across contrasting field and glasshouse environments, providing valuable germplasm for resistance breeding. GWAS identified novel loci associated with resistance; however, most significant SNP associations were trial‐specific and were not consistently reproduced across independent glasshouse experiments. Furthermore, comparison with our previous field‐based GWAS revealed limited overlap between loci identified under glasshouse and field conditions, suggesting that resistance is strongly influenced by environmental conditions. This interpretation is consistent with previous studies showing that resistance phenotypes against *F. graminearum* can differ between greenhouse and field experiments (Hautsalo et al., 2020), while *F. langsethiae* infection and toxin accumulation are highly dependent on environmental conditions (Martin et al., 2018; Gavrilova et al., 2021). The phenotypic variation observed between independent glasshouse trials in the present study, despite identical inoculation protocols, further highlights the environmental sensitivity of the oat–*F. langsethiae* interaction. Together, these findings emphasize the importance of validating resistance loci across multiple environments and years before their application in marker‐assisted breeding. More importantly, our findings highlight a major biological knowledge gap. Despite the economic and food‐safety importance of *F. langsethiae*, the biological mechanisms governing infection, host colonization, and toxin accumulation in oat remain poorly understood. Addressing this biological “black box” will be essential for understanding the environmental drivers of disease development and for developing durable resistance and effective strategies to minimize T‐2 and HT‐2 contamination in oat.

## AUTHOR CONTRIBUTIONS

**Farhana Afroze**: Conceptualization; data curation; formal analysis; writing—original draft; writing—review and editing. **Natarajan Subramani**: Data curation; formal analysis; visualization; writing—original draft; writing—review and editing. **Amal Kahla**: Conceptualization; methodology; writing—review and editing. **Cathal McCabe**: Conceptualization; investigation; writing—review and editing. **Angela Feechan**: Writing—review and editing. **Atikur Rahman**: Conceptualization; investigation; writing—review and editing. **Humberto Fanelli Carvalho**: Data curation; formal analysis; investigation; methodology; writing—review and editing. **Julio Isidro‐Sánchez**: Conceptualization; data curation; formal analysis; investigation; writing—review and editing. **Fiona Doohan**: Conceptualization; funding acquisition; project administration; writing—review and editing.

## CONFLICT OF INTEREST STATEMENT

The author declare no conflicts of interest.

## Supporting information



Supplementary Material

Supplementary Material

Supplementary Material

Supplementary Material

Supplementary Material

Supplementary Material

Supplementary Material

Supplementary Material

Supplementary Material

Supplementary Material

## Data Availability

All data supporting the findings of this study are publicly available in the GitHub repository. Field and glasshouse datasets used in the analysis are available at: https://github.com/NatarajanSubramani/OATGWAS_2026. GWAS summary statistics (including SNP ID, chromosome, position, and *p*‐values for all markers) are available at: https://github.com/TheRocinante‐lab/Publications/tree/main/2026/Subramani_N_et_al_2026
