# Supplementary material for: Identification of germplasm and genomic markers associated with reduced T‐2 and HT‐2 mycotoxin accumulation in oat
Source: Plant Genome. 2026 Oct 9;19(4):e70321. doi: 10.1002/tpg2.70321 (PMC13653033; doi:10.1002/tpg2.70321)
Supplement: Supplementary file 1 [file TPG2-19-e70321-s003.docx]

Table S8. Top BLASTP hits of candidate protein AVESA.00001b.r3.6Ag0003188 (HIPP39) against the NCBI non-redundant (nr) database.

| **Species** | **Protein description** | **Accession** | **Identity (%)** | **Query coverage (%)** | **E-value** |
| --- | --- | --- | --- | --- | --- |
| *Triticum aestivum* | Heavy metal-associated isoprenylated plant protein 39 | XP_044326703.1 | 75.28 | 72 | 4e-39 |
| *Triticum dicoccoides* | HIPP39-like protein | XP_037487826.1 | 74.16 | 72 | 3e-38 |
| *Lolium rigidum* | HIPP39-like protein | XP_047061845.1 | 90.28 | 59 | 8e-38 |
| *Lolium perenne* | HIPP39-like protein | XP_051219495.1 | 90.28 | 59 | 1e-37 |
| *Aegilops geniculata* | hypothetical protein | KAM3391038.1 | 74.16 | 72 | 1e-35 |

BLASTP analysis of the candidate protein (AVESA.00001b.r3.6Ag0003188) was performed against the NCBI non-redundant (nr) protein database, restricted to Poaceae (taxid:4479), including Avena sativa (taxid:4498), Triticum aestivum (taxid:4565), and Hordeum vulgare (taxid:4513). Percent identity, query coverage, and E-values are reported for the top hits.

Table S9. Functional annotation of the candidate heavy metal-associated isoprenylated plant protein (HIPP39) based on UniProt and Barlex databases.

| **Gene/Protein ID** | **Species** | **Database** | **Annotation** | **GO Term (ID)** | **Functional Category** | **Description** |
| --- | --- | --- | --- | --- | --- | --- |
| A0A816XH08 | Barley (*Hordeum vulgare*) | UniProtKB | HMA domain-containing protein | GO:0046872 | Molecular Function | Metal ion binding |
| HORVU.MOREX.r3.1HG0038590.1 | Barley (*Hordeum vulgare*) | Barlex (IPK) | Heavy metal-associated protein | GO:0046872; GO:0030001 | Molecular Function; Biological Process | Metal ion binding; Metal ion transport |
| A0A068L7E9 | Wheat (*Triticum aestivum*) | UniProtKB | Heavy metal-associated isoprenylated protein 3 (HIPP3) | GO:0046872 | Molecular Function | Metal ion binding |

Data were obtained from UniProtKB (<https://www.uniprot.org> ) and the Barlex barley genome database (IPK Gatersleben, Germany; <https://apex.ipk-gatersleben.de/apex/f?p=284> ). Barlex data correspond to the Morex v3 gene model annotation (2021).
