# Supplementary material for: Identification of germplasm and genomic markers associated with reduced T‐2 and HT‐2 mycotoxin accumulation in oat
Source: Plant Genome. 2026 Oct 9;19(4):e70321. doi: 10.1002/tpg2.70321 (PMC13653033; doi:10.1002/tpg2.70321)
Supplement: Supplementary file 2 [file TPG2-19-e70321-s004.docx]

Table S4. Primers used for species-specific qPCR assays

| ***Fusarium* species** | **Forward Primer** | **Reverse Primer** | **Reference** |
| --- | --- | --- | --- |
| *Fusarium Langsethiae* | FlangA29 fwd,  (5′- CAAGTCGACCACTGTGAGTACCTCT-3′) | FlangA95 rev,  (5′- TGTCAAAGCATGTCAGTAAAGATGAC-3′) | (Nicolaisen et al., 2009) |
| *Fusarium sporotrichioides* | FspoA18fwd,  (5′-GCAAGTCGACCACTGTGAGTACA-3′) | FspoA85rev,  (5′-CTGTCAAAGCATGTCAGTAAAAATGAT-3′) | (Nicolaisen et al., 2009) |
| *Avena sativa* | COX554-f (5′-GGTTGTTGCCACCAAGTCTCTT-3′) | COX554-r  (5′-TGCCGCTGCCAACTTC-3′) | (Divon et al., 2011) |

1.Divon, H. H., Razzaghian, J., Udnes-Aamot, H., & Klemsdal, S. S. (2011). Fusarium langsethiae (Torp and Nirenberg), investigation of alternative infection routes in oats. *European Journal of Plant Pathology 2011 132:1*, *132*(1), 147–161. https://doi.org/10.1007/S10658-011-9858-3

2. Nicolaisen, M., Suproniene, S., Nielsen, L. K., Lazzaro, I., Spliid, N. H., & Justesen, A. F. (2009). Real-time PCR for quantification of eleven individual Fusarium species in cereals. *Journal of Microbiological Methods*, *76*(3), 234–240. https://doi.org/10.1016/J.MIMET.2008.10.016
