# Supplementary material for: Identification of germplasm and genomic markers associated with reduced T‐2 and HT‐2 mycotoxin accumulation in oat
Source: Plant Genome. 2026 Oct 9;19(4):e70321. doi: 10.1002/tpg2.70321 (PMC13653033; doi:10.1002/tpg2.70321)
Supplement: Supplementary file 3 [file TPG2-19-e70321-s005.docx]

**List of supplementary materials:**

Figure S1. Relationship between mean T-2+HT-2 toxin concentrations in 11 oat genotypes evaluated under field (natural infection) and glasshouse (artificial *F. langsethiae* inoculation) conditions. Each point represents a genotype mean; the dashed line shows the linear regression fit. The weak, non-significant correlation (Spearman's ρ = 0.17, P= 0.61) indicates inconsistent genotype rankings across environments.

Table S1. List of oat cultivars screened in field trials.

Table S2: List of oat cultivars screened in glasshouse trials.

Table S3. Fungal strains used in this study.

Table S4. Primers used for species-specific qPCR assays

Table S5. Sum of T-2 and HT-2 toxin concentrations (µg kg⁻¹) in oat grain from 190 genotypes under glasshouse conditions, showing individual trial and replicate measurements (n = 4 per genotype). Values ≤20 µg kg⁻¹ represent the assay detection limit.

Table S6. Full GWAS summary statistics, including SNP ID, chromosome, position, p-values, minor allele frequency (MAF), and estimated SNP effects for all markers.

Supplementary File S7. Run-specific GWAS analyses and comparison of association statistics.

Figure S7A. Between-run comparison of genotype mean T-2 + HT-2 toxin concentrations and distributions from the two independent glasshouse experiments. The scatter plot shows the weak agreement between genotype mean toxin concentrations (Pearson r = 0.02; Spearman's ρ = 0.16), while the histograms compare toxin distributions between runs.

Figure S7B. Manhattan and quantile–quantile (QQ) plots from GWAS performed separately for Run 1 and Run 2. Red circles indicate the five SNPs identified in the combined GWAS analysis. These markers were significantly associated with toxin accumulation in Run 2 but not in Run 1.

Table S7. Comparison of association statistics for significant SNP loci identified in the combined and run-specific GWAS analyses, including consensus linkage group (Mrg), physical chromosome position (OT3098 v2), minor allele frequency (MAF), and P-values for the combined analysis, Run 1, and Run 2.

Table S8. Top BLASTP hits of candidate protein AVESA.00001b.r3.6Ag0003188 (HIPP39) against the NCBI non-redundant (nr) database.

Table S9. Functional annotation of the candidate heavy metal-associated isoprenylated plant protein (HIPP39) based on UniProt and Barlex databases.
