# Supplementary material for: Identification of germplasm and genomic markers associated with reduced T‐2 and HT‐2 mycotoxin accumulation in oat
Source: Plant Genome. 2026 Oct 9;19(4):e70321. doi: 10.1002/tpg2.70321 (PMC13653033; doi:10.1002/tpg2.70321)
Supplement: Supplementary file 4 [file TPG2-19-e70321-s007.docx]

Table S3. Fungal strains used in this study.

| **Species** | **Strains** | **Country of origin** | **Source** |
| --- | --- | --- | --- |
| *Fusarium langsethiae* | Fl201059 | UK | NIBIO, Norway |
| *F. poae* | 202172 | Norway | NIBIO, Norway |
| *F. Sporotrichioides* | 89/N | Ireland | SBES, UCD^*^ |

**UCD = University College Dublin. Strain Fl201059 was used for glasshouse inoculations and to generate standard curves for *F. langsethiae* qPCR quantification; strain 89/N was used for standard curves and as a positive control for *F. sporotrichioides* PCR. All strains are available upon request, subject to institutional agreements.
