# Supplementary material for: Identification of germplasm and genomic markers associated with reduced T‐2 and HT‐2 mycotoxin accumulation in oat
Source: Plant Genome. 2026 Oct 9;19(4):e70321. doi: 10.1002/tpg2.70321 (PMC13653033; doi:10.1002/tpg2.70321)
Supplement: Supplementary file 5 [file TPG2-19-e70321-s009.docx]

Table S1. List of oat cultivars screened in field trials.

| **Cultivar Name** | **Seed Source** | | **Release date** | **Origin** |
| --- | --- | --- | --- | --- |
| 021-SO2011Au16 | Aberystwyth University, Wales | | Unknown | United Kingdom |
| Barra^1^ | University College Dublin | | Unknown | Sweden |
| Blythe | Aberystwyth University, Wales | | Unknown | United Kingdom |
| Caron | Aberystwyth University, Wales | | 1983 | United Kingdom |
| Conway^1^ | Aberystwyth University, Wales | | Unknown | United Kingdom |
| Elen | Aberystwyth University, Wales | 1980 | | United Kingdom |
| Glasnevin Major^1^ | University College Dublin | 1930 | | Ireland |
| Hedvig^1^ | University College Dublin | 1981 | | Sweden |
| JOKIKYLÄ ME0501 | University College Dublin | Unknown | | Sweden |
| Lena^1^ | University College Dublin | 1996 | | Norway |
| Menai | Aberystwyth University, Wales | 1979 | | United Kingdom |
| Milford | Aberystwyth University, Wales | 1943 | | United Kingdom |
| Milo | Aberystwyth University, Wales | 1980 | | United Kingdom |
| Poretto^1^ | University College Dublin | 2004 | | Netherlands |
| Sandy^1^ | University College Dublin | 1850 | | United Kingdom |
| Stormont Arrow^1^ | University College Dublin | 1938 | | Ireland |
| SW Betania^1^ | University College Dublin | 2006 | | Sweden |
| Tempo^1^ | University College Dublin | 1950 | | Norway |
| Timpani | Aberystwyth University, Wales | Unknown | | United Kingdom |

^1^These cultivars were evaluated in both field and glasshouse experiments. All other cultivars were evaluated in field studies.
