# Supplementary material for: Identification of germplasm and genomic markers associated with reduced T‐2 and HT‐2 mycotoxin accumulation in oat
Source: Plant Genome. 2026 Oct 9;19(4):e70321. doi: 10.1002/tpg2.70321 (PMC13653033; doi:10.1002/tpg2.70321)
Supplement: Supplementary file 6 [file TPG2-19-e70321-s010.docx]

**Supplementary Figure S7A.** Between-run comparison of genotype mean T-2 + HT-2 toxin concentrations (left) and distributions of genotype mean toxin concentrations for Run 1 and Run 2 (right). The scatter plot shows the weak agreement between genotype means across the two independent glasshouse experiments (Pearson *r* = 0.02; Spearman ρ = 0.16), while the histograms illustrate the differences in toxin distributions between runs.

**Supplementary Figure S7B.** Manhattan and quantile–quantile (QQ) plots from GWAS performed separately for Run 1 and Run 2. Red circles indicate the five SNPs identified in the combined analysis. These markers were strongly associated with toxin accumulation in Run 2 but were not significantly associated in Run 1, consistent with the weak agreement between runs.

Supplementary Table S7. Comparison of association statistics for significant SNP loci identified in the combined and run-specific GWAS analyses.

| **Locus / SNP** | **Mrg / cM** | **MAF** | **OT3098 v2 chr** | **OT3098 v2 bp** | **Comb / Run1 / Run2 p** |
| --- | --- | --- | --- | --- | --- |
| Locus 1 (11885 ×3) | Mrg11 / 3.7 | 0.021 | 6A & 4C* | 6A:432,536,024; 4C:2,739,319 | 3.0e-5 / 0.48 / 2.5e-7 |
| Locus 2 (13857.17) | Mrg11 / 9.8 | 0.047 | 4C | 21,540,460 | 7.8e-5 / 0.41 / 2.6e-6 |
| Locus 2 (39187.31) | Mrg11 / 9.8 | 0.051 | 4C | 21,549,717 | 7.8e-5 / 0.46 / 1.7e-6 |

Note: The avgbs_cluster_11885 marker represents a duplicated (homoeologous) GBS tag that aligns equally well to chromosomes 6A and 4C in the OT3098 v2 assembly. The chromosome 6A position is used in the main manuscript because it contains the positional candidate gene HIPP39.
